# Oral Biofilm Architecture on Natural Teeth

**DOI:** 10.1371/journal.pone.0009321

**Published:** 2010-02-24

**Authors:** Vincent Zijnge, M. Barbara M. van Leeuwen, John E. Degener, Frank Abbas, Thomas Thurnheer, Rudolf Gmür, Hermie J. M. Harmsen

**Affiliations:** 1 Center for Dentistry and Oral Hygiene, University of Groningen, Groningen, The Netherlands; 2 Department of Medical Microbiology, University of Groningen, Groningen, The Netherlands; 3 Department of Biomedical Engineering, University of Groningen, Groningen, The Netherlands; 4 Institute of Oral Biology, University of Zürich, Zürich, Switzerland; Charité-Universitätsmedizin Berlin, Germany

## Abstract

Periodontitis and caries are infectious diseases of the oral cavity in which oral biofilms play a causative role. Moreover, oral biofilms are widely studied as model systems for bacterial adhesion, biofilm development, and biofilm resistance to antibiotics, due to their widespread presence and accessibility. Despite descriptions of initial plaque formation on the tooth surface, studies on mature plaque and plaque structure below the gum are limited to landmark studies from the 1970s, without appreciating the breadth of microbial diversity in the plaque. We used fluorescent in situ hybridization to localize in vivo the most abundant species from different phyla and species associated with periodontitis on seven embedded teeth obtained from four different subjects. The data showed convincingly the dominance of *Actinomyces* sp., *Tannerella forsythia*, *Fusobacterium nucleatum*, *Spirochaetes*, and *Synergistetes* in subgingival plaque. The latter proved to be new with a possibly important role in host-pathogen interaction due to its localization in close proximity to immune cells. The present study identified for the first time in vivo that *Lactobacillus* sp. are the central cells of bacterial aggregates in subgingival plaque, and that *Streptococcus* sp. and the yeast *Candida albicans* form corncob structures in supragingival plaque. Finally, periodontal pathogens colonize already formed biofilms and form microcolonies therein. These in vivo observations on oral biofilms provide a clear vision on biofilm architecture and the spatial distribution of predominant species.

## Introduction

Oral microbial biofilms are three-dimensional structured bacterial communities [1] attached to a solid surface like the enamel of the teeth, the surface of the root or dental implants [2] and are embedded in an exo-polysaccharide matrix [3]. Oral biofilms are exemplary and served as a model system for bacterial adhesion [4], [5] and antibiotic resistance [6].

The appreciation of the complex nature of oral biofilms was highlighted decades ago by the work of Listgarten and co-workers who described the architecture of biofilms by light and electron microscopy on epoxy resin crowns and extracted teeth [7], [8]. Supragingivally, on the enamel, they observed the formation of columnar micro-colonies with their long axis perpendicular to the crown surface. Gram-positive cocci dominated these columns and occasionally, some isolated branching filaments were found after one day of growth. After one week filaments appeared on top of the columns. After three weeks, the biofilm was predominantly filamentous without any sign of cocci left. Filaments seemed to have colonized and subsequently replaced the predominantly coccoid population. A loose layer of so-called corncobs covered the three-week-old biofilm. Corncobs were thought to be bacterial aggregates with a central filamentous cell surrounded by cocci attached to it. After two months, the general features of the biofilm resembled those found at the three weeks time point. Most noticeably was the gingival area, where a fuzzy layer of spirochetes covered the biofilm. This fuzzy layer contained bacterial aggregates resembling test-tube brushes. There were rough and fine types of these brushes. In a study examining biofilm structure at varying degrees of periodontal health, the gingivitis and periodontitis associated biofilms resembled largely the two months old plaque on epoxy resin crowns. Filamentous bacteria were predominant in the biofilm. Between the adhered biofilm and the soft tissue of the pocket, a layer without a well-defined extracellular matrix was observed. This layer consisted of spirochetes, flagellated bacteria and test-tube brushes [8]. The major hindrance of these electron microscopy studies was the inability to identify the species in the biofilm, corncobs or test-tube brushes.

**Figure 1:**
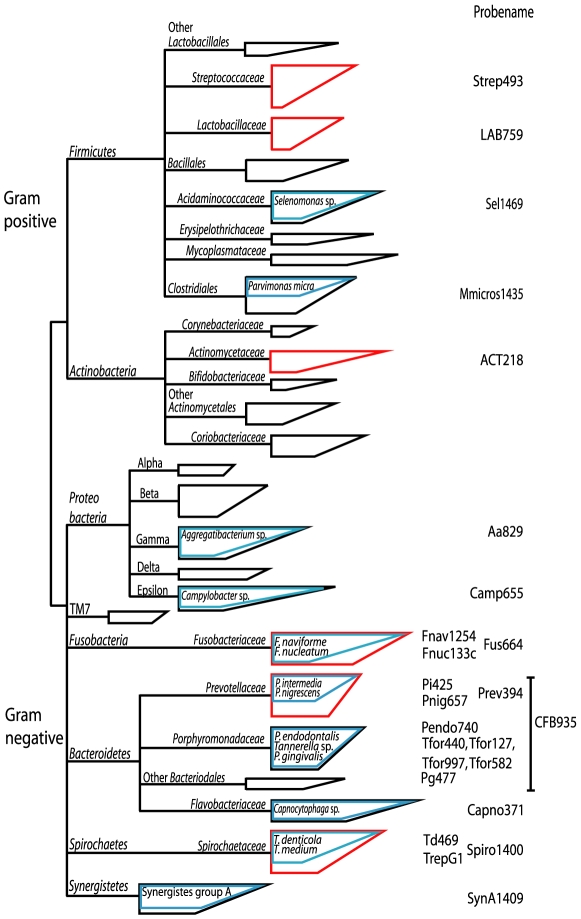
Phylogenetic tree representing oral microbial diversity. The tree is based on >1500 sequences derived from oral-cavity studies and shows the schematic coverage of the diversity by our probe set. The branching of the tree was simplified for clarity. The boxes represent groups of bacteria. The vertical size of the box reflect the number of sequences and the angular side the genetic diversity. The blue boxes indicate the part that is covered by the species or subgroup-specific probes and the red box indicate genus-specific probes.

Using fluorescent *in situ* hybridization (FISH), it was shown for the first time *in vivo* that initial biofilm formation was the result of co aggregation and adhesion between *Streptococcus* spp. and *Actinomyces* spp. [9]. In a later study with the same technique, it was shown *in vivo*, that after seven days the proportion of streptococci decreased and the proportion of *Fusobacterium nucleatum* increased [10]. Subgingival biofilms formed on expanded polytetrafluoroethylene carriers that had been inserted into the depth of periodontal pockets have been studied with FISH with only two probes, one with specificity for a large group of oral treponemes and the other recognizing all oral bacteria [11]. The bacterial diversity in the oral cavity is estimated to be more than 700 different species and phylotypes, belonging to nine phyla; *Deferribacteres*, *Spirochaetes*, *Fusobacteria*, *Actinobacteria*, *Firmicutes*, *Bacteroidetes*, *Proteobacteria* and two phyla without cultiviable members; OP11 and TM7, which is summarized in Figure 1. Little is known about the spatial distribution of these taxa in oral biofilms. The aim of the present study therefore was to reveal the *in vivo* architecture of supra and subgingival plaque with a panel of 16S or 18S rRNA targeted FISH-probes covering the most important groups of oral microorganisms, and to provide an essential step from oral microbial diversity to oral biofilm function.

## Results

From 10 examined teeth, seven showed a positive signal after hybridization with fluorescently labeled probes. Macroscopic analysis of Gram-stained sections revealed the localization of the plaque in relation to the cemento-enamel junction and gingival tissue. A phylogenetic tree based on approximately 1500 nearly complete (>1300 bp) sequences was constructed. Sequences were derived from molecular studies of oral microbial diversity and a manual search through the SILVA database [12]. The coverage of the applied probes is represented in Figure 1. It shows that a representative part of the oral microbial diversity is covered.

### Subgingival Biofilm Architecture

**Figure 2:**
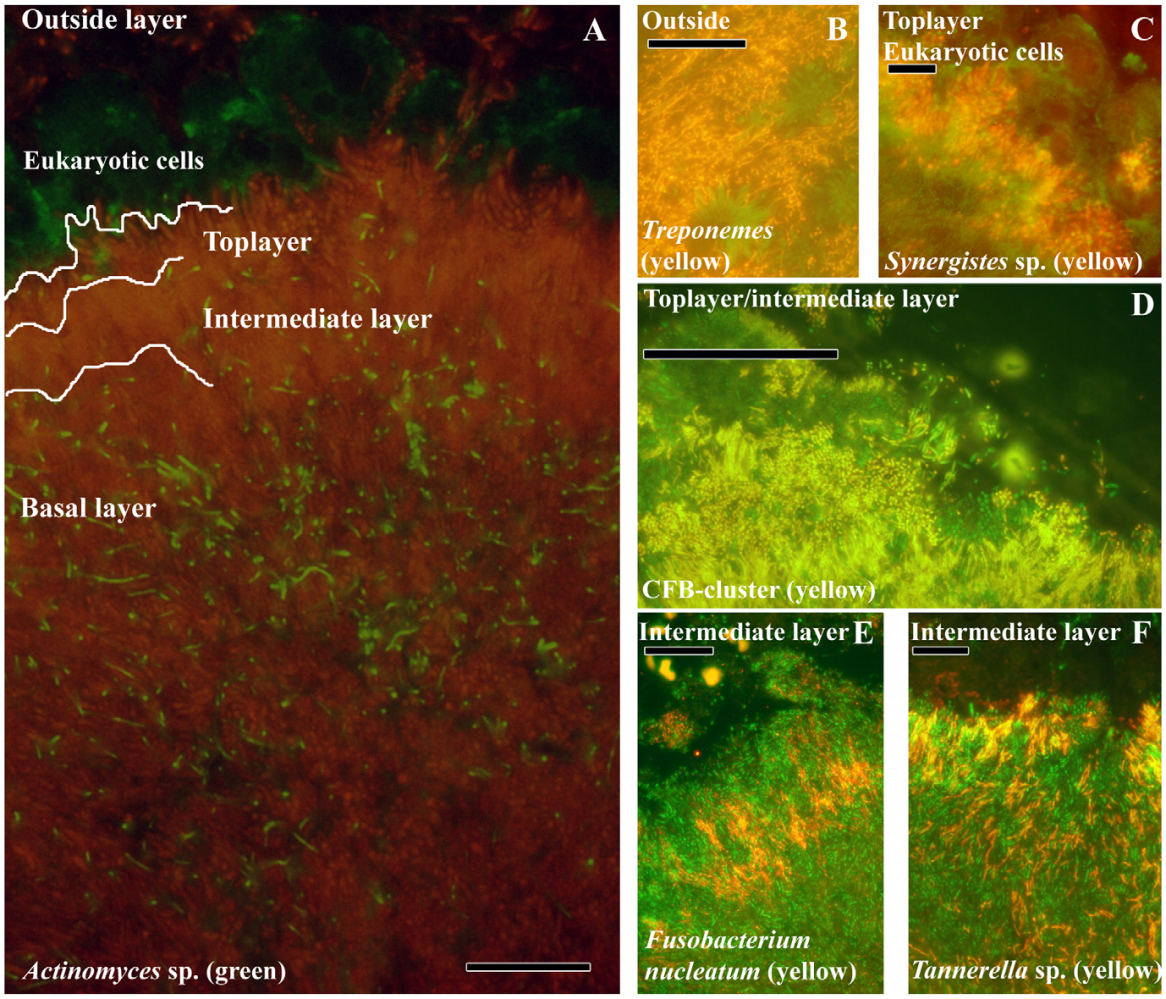
Localization of the most abundant species in subgingival biofilms. (A) Overview of the subgingival biofilm with *Actinomyces* sp. (green bacteria), bacteria (red) and eukaryotic cells (large green cells on top). (B) *Spirochaetes* (yellow) outside the biofilm. (C) Detail of *Synergistetes* (yellow) in the top layer in close proximity to eukaryotic cells (green). (D) CFB-cluster (yellow) in the top and intermediate layer. (E) *F. nucleatum* in the intermediate layer. (F) *Tannerella* sp. (yellow) in the intermediate layer. Each panel is double-stained with probe EUB338 labeled with FITC or Cy3. The yellow color results from the simultaneous staning with FITC and Cy3 labeled probes. Bars are 10 µm.

The localization of the most abundant subgingival bacteria is summarized in Figure 2. Panel A shows a typical subgingival biofilm with increasing fluorescent intensity from the tooth side towards the epithelium side. Based on differences in bacterial morphologies and fluorescence intensities, four different layers were distinguished. The first layer of the biofilm is composed of cells displaying little fluorescence relative to cells in the top of the biofilm. Of all the probes tested, only *Actinomyces* sp. gave a positive signal in this layer. The intermediate layer is composed of many spindle-shaped cells of which *F. nucleatum*, *T. forsythia* and possibly other *Tannerella* sp. positive with probe Tfor127 are visible as the red/yellow band of bacteria in panels E and F of Figure 2. The top layer of the biofilm and part of the intermediate layer contain mainly bacteria belonging to the *Cytophaga-Flavobacterium-Bacteroides* cluster (CFB-cluster) as detected with probe CFB935 and shown in panel D. CFB935 positive cells are filamentous, rod-shaped or even coccoid. Samples double stained with CFB935 and *Tannerella*-specific probes showed that most filamentous bacteria are *Tannerella* sp., while many of the rod-shaped bacteria are *Prevotella* sp. and *Bacteroidetes* species as detected with the group-specific probes PREV392 and BAC303, respectively. Besides the presence of bacteria from the CFB-cluster, large cigar-like bacteria were in the top layer. These cells belong to the *Synergistetes* group A of bacteria and form a ‘palisade’-like lining. They were in close contact to eukaryotic cells resembling polymorphonuclear leukocytes (PMN's) according to the presence of polymorph nuclei (panel C). Outside the biofilm, a fourth layer without clear organization was observed. *Spirochaetes* were primary localized in the fourth layer where they are the most dominant species. Bacterial aggregates, called rough and fine test-tube brushes were detected between the *Spirochaetes* (Panel B).

### Supragingival Biofilm Architecture

**Figure 3:**
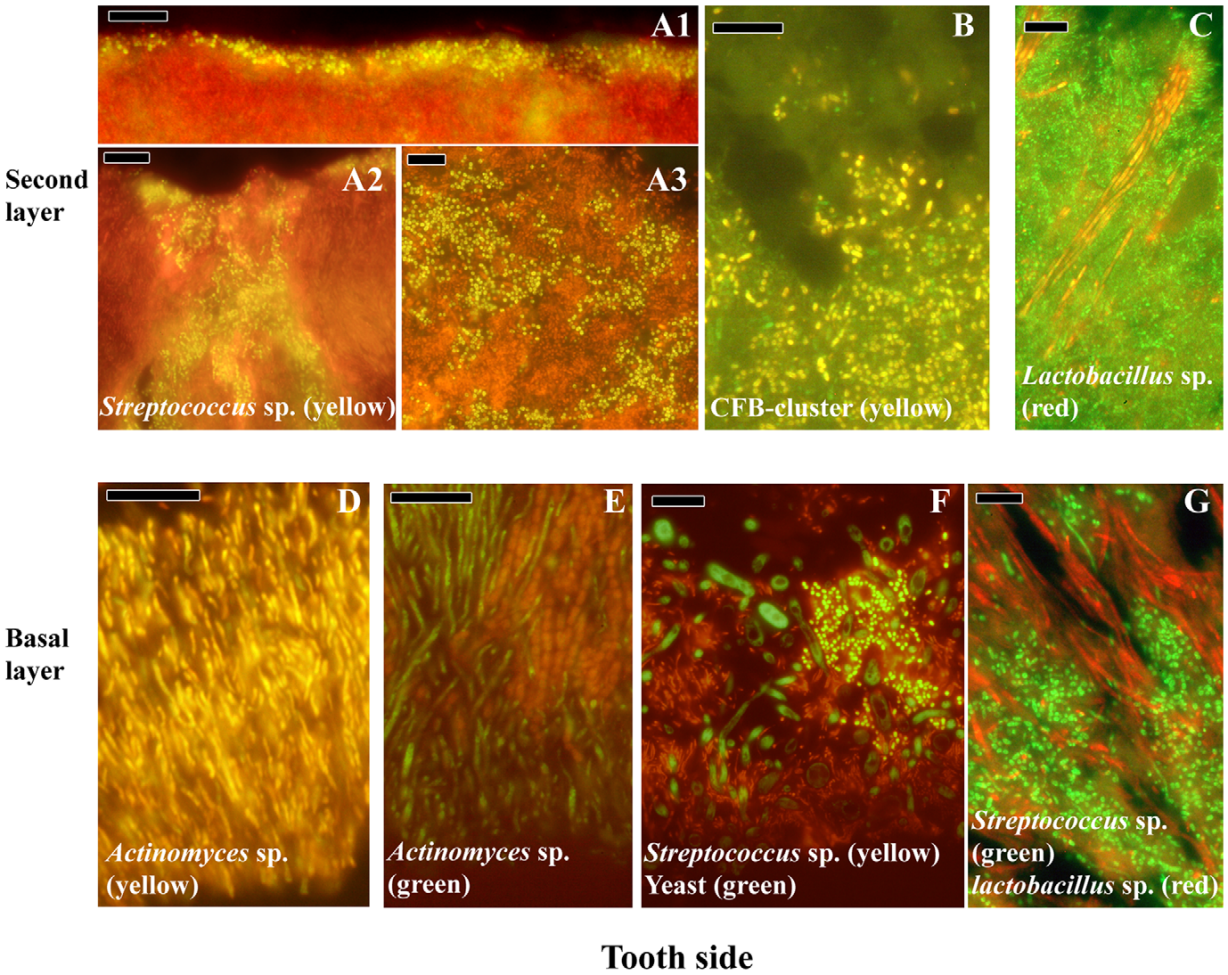
Localization of the most abundant species in supragingival biofilms. *Streptococcus* sp. (yellow) form a thin band on top of the biofilm (A1), almost engulfing in the biofilm (A2) or present as small cells scattered through the top layer of the biofilm (A3). (B) Cells from the CFB-cluster of bacteria in the top layer of the biofilm, without defined structure. (C) *Lactobacillus* sp. (red) forming long strings through the top layer. (D) *Actinomyces* sp. (yellow) plaque attached to the tooth. (E) *Actinomyces* sp. (green) and cocci forming initial plaque.(F) Multispecies initial plaque composed of *Streptococcus* sp. (yellow), yeast cells (green) and bacteria unidentified (red). (G) *Streptococcus* sp. (green) and *Lactobacillus* sp. (red) forming initial plaque. Black holes might be channels through the biofilm. Panels A, B, C, E, F are double stained with probe EUB338 labeled with FITC or Cy3. Bars are 10 µm.

Supragingival biofilms are more heterogeneous in architecture compared to subgingival biofilms. In general, two different layers could be observed. The basal layer adheres to the tooth surface and four different biofilm types were observed (Figure 3). First, a biofilm composed of only rod shaped *Actinomyces* cells perpendicularly orientated to the tooth surface (panel D). The second type is a mixture of *Actinomyces* sp. and chains of cocci, not identified as streptococci, perpendicularly orientated to the tooth surface (panel E). The third type shows a biofilm with filamentous bacteria, streptococci and yeasts, where streptococci form a distinct colony around yeast cells (panel F). The fourth type is a biofilm composed of mainly streptococci growing in close proximity to *Lactobacillus* sp. that are orientated perpendicularly to the tooth surface (panel G).

The second layer can be found on top of any biofilm type of the basal layer. *Streptococcus* sp. can be present as heterogeneously scattered cells through the second layer of the biofilm without any apparent organization (panel A3), or they can be aligned on top of the second biofilm layer as a thin coat (panel A1). In addition, they colonize cracks in the biofilm (panel A2). Also, there is a heterogeneous scattering of bacterial cells belonging to the CFB-cluster (panel B). Finally, *Lactobacillus* sp. that are orientated away from the tooth surface are surrounded by cells with different morphologies (panel C).

### Subgingival Localization of Periodontal Pathogens

**Figure 4:**
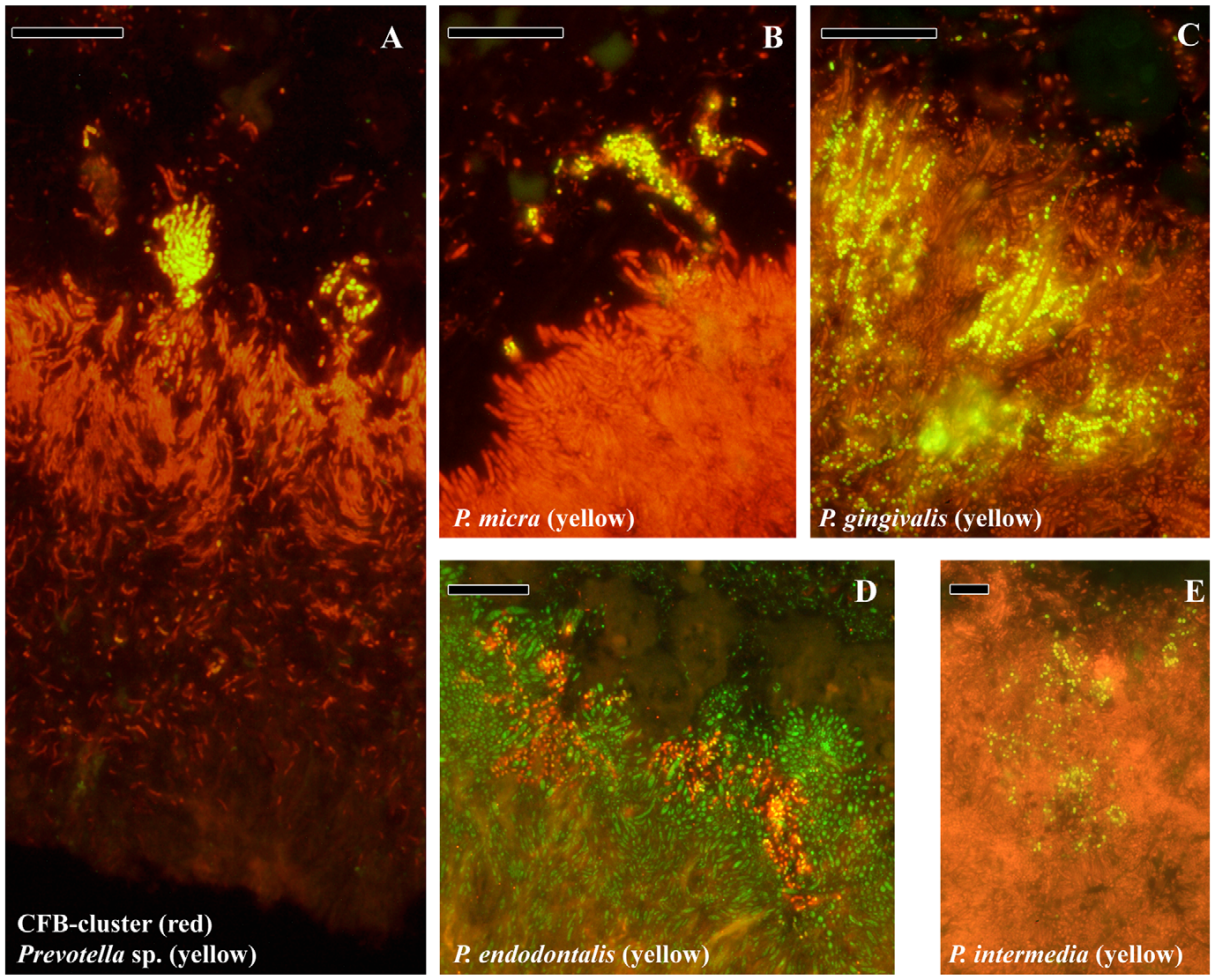
Localization of species associated with periodontitis. (A) Overview of the subgingival biofilm with CFB-cluster species (red) and *Prevotella* sp. (yellow). Since *Prevotella* sp. are part of the CFB-cluster of bacteria, cells appear in yellow. (B) Top of the biofilm with a micro-colony of *P. micra* (yellow). (C) Micro-colonies of *P. gingivalis* (yellow) in the top layer. (D) Micro-colonies of *P. endodontalis* (yellow) in the top layer. (E) Micro-colonies of *P. intermedia* in the top layer. Panels B, C, D and E are double stained with probe EUB338 labeled with FITC or Cy3. Bars are 10 µm.

The localization of presumptive periodontal pathogens in the subgingival biofilm is shown in Figure 4. Most of the periodontal pathogens belong to the gram-negative group of bacteria united in the CFB-cluster like *P. gingivalis*, *P. intermedia*, *P. endodontalis* or *P.nigrescens*. Most CFB-cluster cells are evenly distributed in the top and intermediate layer of the biofilm. *Prevotella* sp. however, colonize the biofilm in micro-colonies (panel A) which sometimes are located on top of the biofilm, but, as is shown for *P. intermedia*, also reside within the top layer (panel E). *Porphyromonas gingivalis* and *Porphyromonas endodontalis* appear mainly as micro-colonies within the top layer (panel C and D). *Parvimonas micra*, an example of a gram-positive species that is associated with periodontitis, is also found in micro-colonies in the top layer (panel B). Apparently, the microorganisms considered pathogens are mostly present in micro-colonies in the top layer and in the fourth layer of the subgingival biofilm or can be part of bacterial aggregates.

### Bacterial Aggregates or Structures in Dental Plaque

**Figure 5:**
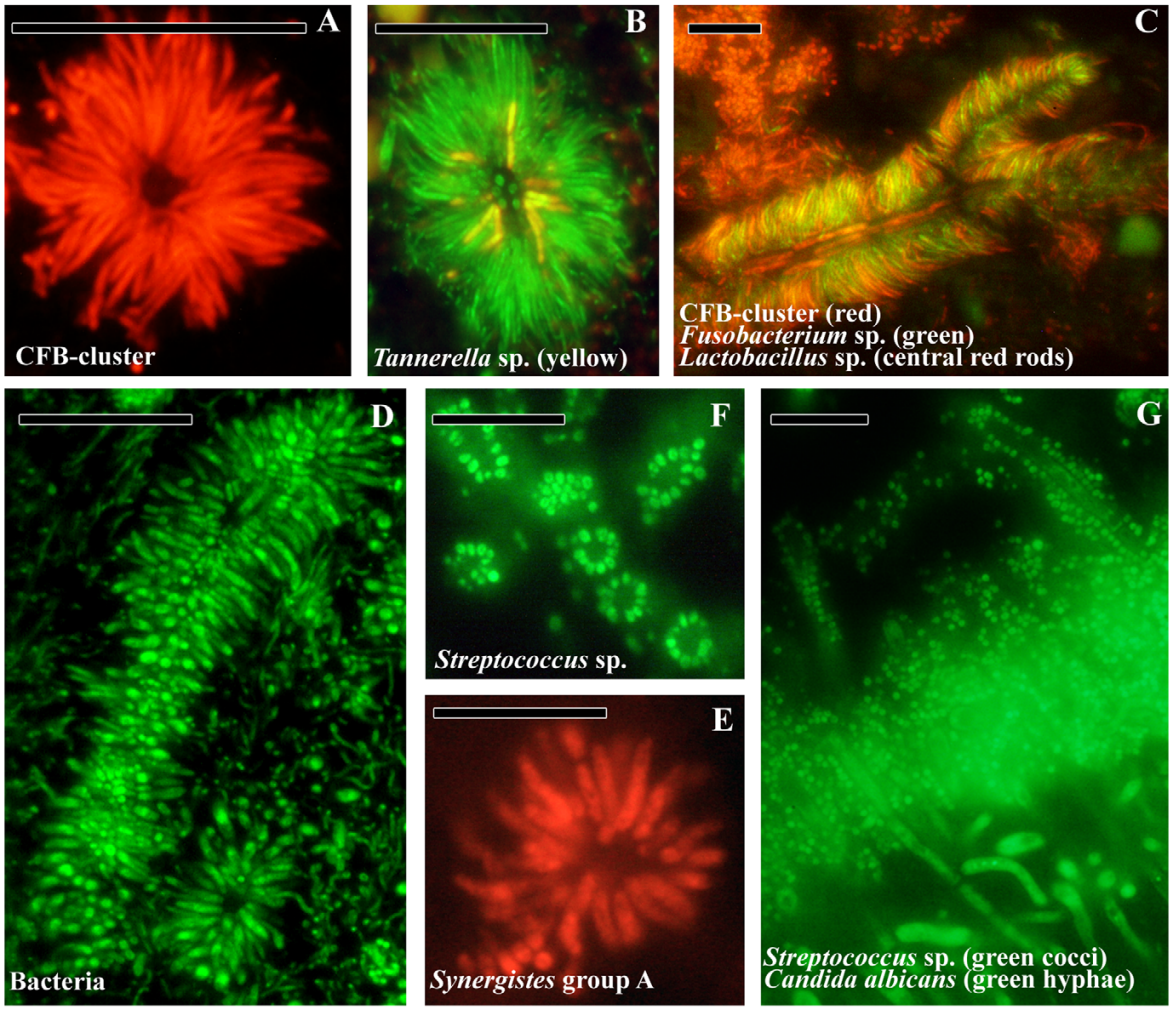
Bacterial aggregates in oral plaque. (A) Transversal view of a test-tube brush found in subgingival plaque composed of filamentous cells from the CFB-cluster. (B) *Tannerella* sp. (yellow) in a test-tube brush. (C) Longitudinal view of a test-tube brush with *Lactobacillus* sp. (red rods) as central structures. *F. nucleatum* (green) and CFB-cluster filaments radiating from the central structures. (D) Longitudinal and transversal view of a test-tube brush stained with the eubacterial probe. (E) Transversal view of the test-tube brush in panel D, composed of *Synergistetes* group A species. (F) Transversal view of *Streptococcus* sp. (green) aggregation around a central cell (not stained) in supragingival plaque. (G) Transversal view of supragingival plaque with *Streptococcus* sp. (green cocci) and *Candida albicans* (green hyphae) in the top layer of the biofilm and forming corn cob structures growing outwards. Bars are 10 µm.

Aggregates of microorganisms have been detected in both sub and supragingival plaque (Figure 5). In line with previous reports, different aggregate morphologies were observed in the fourth layer of subgingival plaque. Filaments from the CFB-cluster, morphologically like *T. forsythia*, and *F. nucleatum* are arranged perpendicularly around lactobacilli, forming fine test-tube brushes (panel A–C). There have also been observations of test-tube brushes composed of a complex mixture of cells with *T. forsythia*, *Campylobacter* sp., *P. micra*, *Fusobacteria* and *Synergistetes* group A, among others. *Synergistetes* cells may also form aggregates exclusively with themselves (panel D and E). In supragingival plaque, corncob structures consist of streptococci adhering to a central axis of yeast cells or hyphae.

## Discussion

The aim of the present study was to unravel tooth attached biofilm architecture. For the first time, bacteria in these biofilms are identified and localized *in vivo* using FISH. The results present new insights into the architecture of tooth-attached biofilms and visualize the interaction of the biofilm with the human immune system. FISH offers the opportunity to obtain positional information of bacteria in intact biofilms, its application overcomes the limits of culturability and can relatively easily be extended to new identified species and phylotypes. *Synergistetes* sp. for example, only recently gained attention [13] but may account for 3–11% of the bacterial population [14]. In addition, they form a palisade-like lining along the outer length of the biofilm and were in direct contact with host immune cells suggesting an important role in host-biofilm interactions.

In our current experimental design, group-level probes and species-specific probes were applied to efficiently identify cells that might be of interest due to their location in the biofilm, this was seen with *Lactobacillus* sp. as the central axis of test-tube brushes and streptococci and Candida as species that form corncobs in supragingival plaque.

Understanding the role of microorganisms in oral diseases needs a unifying concept incorporating biofilm diversity, structure and function. A first oral biofilm model was based on co-aggregation experiments [15]. The model presents a final composition of the biofilm, without taking into consideration the spatio-temporal dynamics of biofilm formation. The figures of the present study are composed of consistent observations from hundreds of thin sections from seven different teeth of four different persons. Each observation provides only a “snap shot” of plaque architecture without quantitative measurements or dynamic time lap observations. Combining multiple observations of supra- and subgingival biofilms reflects to some degree the dynamics of biofilm formation, which leads us to the following view of plaque formation.

Initial plaque formation starts with the deposition of a salivary pellicle on the tooth surface. Planktonic cells or aggregates of cells adhere to this pellicle via specialized adhesins on the bacterial cell surface that recognize pellicle proteins [16] and by non-specific physico-chemical interactions [5]. These phenomena may result in a scattered pattern of bacterial deposits [9], [17] composed of initial colonizers like *Actinomyces* sp., *Streptococcus* sp., *Lactobacillus* sp. and *Candida* sp. [18]–[20] and is reflected in the different biofilm types of the first layer of supragingival plaque (Figure 3). Maturation of the biofilm proceeds via co-aggregation of planktonic bacteria to the already adhered biofilm [21] and bacterial growth, as has been shown for *Streptococcus sanguinis* [22]. The second layer may be the result of both processes. The presence of either *Streptococcus* sp. or bacteria from the CFB-cluster in the second layer of Figure 3 may reflect a crucial transition in supragingival plaque from a predominantly gram-positive saccharolytic plaque to a gram-negative proteolytic plaque that might be the result of the availability of nutrients *e.g.* dietary sugars or proteins from saliva and crevicular fluids. It was noticed that after three weeks, undisturbed supragingival plaque morphologically resembled subgingival plaque [7]. In our observations of subgingival plaque, the fluorescence intensity of the bacterial cells stained with the eubacterial probe increased from the tooth side towards the epithelium. This reflects differences in physiological activity of the cells [23]. In the basal layer of Figure 2, only *Actinomyces* sp. showed positive of all the probes tested. The unidentified cells may belong to new species for which no probes have been developed. Another explanation might be that the basal layer constitutes previous stages of the biofilm that have become secluded from nutrients and contain dead or physiologically inactive cells with lower fluorescence intensity as has been shown *in vitro* [24], [25]. Of the initial colonizers, only *Actinomyces* sp. might survive, maybe due to their capacity to store intracellular glycogen [26] or their capacity to scavenge on biofilm material like extracellular polymeric substances and on compounds from dead bacterial cells. These are the first *in vivo* observations of graduated differences in the physiological activity of cells within the biofilm, and support the idea that bacterial growth is an important determent of oral biofilm development [27].

In the intermediate layer, *T. forsythia* may benefit from its close proximity to dead cells in the basal layer. These may serve as a source of exogenous N-acetyl-muramic acid, a bacterial cell wall sugar on which *T. forsythia* is dependent [28]. In the presence of *F. nucleatum*, *T. forsythia* synergistically forms robust biofilms via cell-cell contacts *in vitro* [29] as is reflected in their prominent and abundant co-localization of both species along the entire length of the biofilm. The presence of *F. nucleatum* in the intermediate layer, as proposed by Kolenbrander and London [15], is confirmed for the first time *in vivo* in the present study. These structural observations on the dominance of *Actinomyces* sp., *Fusobacteria* and *T. forsythia* are supported by dot-blot analysis of subgingival plaque [30]. In contrast, *P. gingivalis*, *P. intermedia*, *P. endodontalis* and *P. micra* are mainly located in the top layer of the biofilm in micro-colonies [31], [32]. The presence of micro-colonies proposes a distinction between species that are structurally present, probably forming the framework of the biofilm, and transient species that can colonize the already established biofilm forming micro-colonies.

Summarizing, the present study on oral biofilms links early studies on biofilm structure and recent molecular insights in oral bacterial diversity. This resulted in important new observations on oral biofilms, in architecture and in dynamics. First, the species that form test-tube brushes and corncobs are identified for the first time *in vivo*. Second, the localization of *T. forsythia* in the intermediate layer of oral biofilms should be incorporated in the biofilm model, as well as a fourth layer of unattached plaque consisting of mainly *Spirochaetes*. Third, the observation of bacteria that are either structural members of the subgingival biofilm, e.g. *Actinomyces*, *Fusobacteria*, *Tannerella* sp., or species that colonize an already formed biofilm, e.g. *P. intermedia*, *P. gingivalis* and *P. micra*. Fourth, the biofilm model based on co-aggregation should include bacterial growth and appreciate the dynamics of biofilm maturation. Finally, the finding of a palisade lining of *Synergistetes* sp. in the close proximity to host defence cells suggests a major role in host biofilm interactions. These results provide an oral biofilm model and show that *in vivo* observations on biofilm architecture are an essential link between molecular diversity and bacterial function in relation to oral diseases.

## Materials and Methods

### Ethics Statement

All protocols were approved by the Medical Ethical board of the University Medical Center Groningen. Extracted teeth were collected as anonymous by-products of regular treatment. As such, the Medical Ethical board stated that the performed research was not conducted under the regulations of the Act on Medical Research Involving Human Subjects (METc 2009.305). A written informed consent was therefore not compulsory. Nevertheless, patients were informed about the research purposes and gave verbal informed consent, which was not recorded to keep the procedure anonymously.

### Sample Collection and Handling

Ten teeth from four patients were used in this study. The patients were referred to the Dept. of Oral Surgery and Periodontology for extraction of their remaining teeth and the fabrication of complete dentures. Teeth were diagnosed with advanced generalized periodontitis based on pocket depth recordings of >6 mm and x-rays indicating more than 30% bone loss. Subjects had not taken antibiotics within the last three months and did not suffer from systemic diseases. An experienced dentist carefully extracted the teeth without the use of elevators, not to disturb the subgingival plaque. Immediately after extraction, the teeth were placed in 3% (wt/vol) paraformaldehyde (PFA) in phosphate-buffered saline (PBS) (8 g of NaCl, 0.2 g of KCl, 1.44 g of Na_2_HPO_4_ and 0.24 g of KH_2_PO_4_ per liter; pH 7.2) and fixed at 4°C for 16 h. Fixed teeth were dehydrated in 50%, 60%, 70%, 80%, 90% and 100% (vol/vol) ethanol-PBS in subsequent sessions of 1 hour. Teeth were either stored in 60% (vol/vol) ethanol-PBS at −20°C until further use or processed immediately.

### Sample Processing

Fixed and dyhydrated teeth were carefully embedded in Technovit 8100 (Heraeus Kultzer GmbH) at 4°C. The embedded tissue was either cut transversal or a combination of longitudinal and transversal (50/50) in cross-sections of 1 mm with a water-cooled rotary saw. The embedded cross-sections were decalcified with a 17% ethylene-diamine-tetraacetic acid decalcifying solution (pH 7.0), which was renewed regularly during the course of decalcification, varying in duration from 16 to 22 days depending on the size of the specimens. Regular x-ray analysis confirmed completion of decalcification. Decalcified cross-sections were re-embedded in Technovit 8100 and stored at 4°C. Sections of two micron were obtained with a Tungsten carbide knife in a rotary microtome (Reichart-Jung) and stretched on water. Stretched sections were mounted to polysine precoated glass slides (Thermo Scientific) for FISH analysis.

### FISH Analysis

Oligonucleotide probes, labeled at the 5′- and 3′ end with fluorescein (FITC) or at the 5′- end with Cy3 were purchased from Eurogentec (Eurogentec, Maastricht, the Netherlands). A set of 29 FISH probes, specific at the domain or group level were used together with species-specific probes (Table 1). The target bacteria of probe LAB759 needed pre-treatment with Labmix (25 mM Tris-HCl pH 7.5, 10 mM EDTA, 585 mM sucrose, 5 mM CaCl_2_, 0.3 mg/ml sodiumtaurocholate, 0.1 mg/ml lipase and 2 mg/ml lysozyme) for 1 h at 37°C. To enable probe penetration, other gram-positive targets needed lysozyme pre-treatment for 15 min at room temperature with lysozyme buffer (2 mg/ml lysozyme, 100 mM Tris-HCl, pH 8.0) as indicated in Table 1. Standard FISH procedures were followed with a hybridization time of three hours and formamide concentration and hybridization temperature (46°C or 50°C) according to the references (Table 1), with the aim of achieving optimal stringency and specificity. The biofilms were examined using a Leica DM RXA microscope (Leica Mikroskopie). Filters were set to 500–540 nm for FITC and 570–630 nm for Cy3. Images were obtained using 63× (numeric aperture 1.0) oil immersion objectives. Color micrographs were taken with a digital Canon EOS400 Camera, transferred to an HP personal computer and processed using Photoshop 6.0 (Adobe) without any qualitative changes to the raw images.

**Table 1:** Oligonucleotide probe sequences, their targets and the hybridization conditions used in this study.

Target	Probename	Label	Sequence 5′→3′	T_m_ (°C)	Pretreatment	Formamide (%)	Hyb. Temp. (°C)	Reference
most Bacteria	EUB338	FITC/Cy3	GCT GCC TCC CGT AGG AGT	55	No	0–50	50	[33]
Eukarya	EUK502	FITC	ACC AGA CTT GCC CTC C	52	No	15–45	50	[33]
*Candida albicans*	CAAL	Cy3	GCC AAG GCT TAT ACT CGC T	51	No	30	50	[34]
most *Streptococcus* spp. and some *Lactococcus* spp.	STR493	FITC	GTT AGC CGT CCC TTT CTG G	53	15 min 37°C lysis buffer	0	50	[35]
*Lactobacillus* sp., *Ruminococcaceae* sp. and *Pediococcus* sp.	LAB759	Cy3	CTA CCC ATR CTT TCG AGC C	59	60 min 37°C labmix	35	46	This study
*Parvimonas micra*	Mmicros1435	FITC	GCC GCC GAT CTA ACC GCA	64	No	0	50	[36]
*Selenomonas* sp.but not *S. sputigena*	Sel1469	Cy3	CCA GTC ACC TTC CCC ACC	58	No	30–50	46	This study
*Synergistetes group A*	SynA1409	Cy3	ACA CCC GGC TCG GGT GGT	62	No	40–50	46	This study
*Actinomyces* sp.	ACT218	FITC	CGA GCC CAT CCC CCA CCA	57	15 min 37°C lysis buffer	0	50	This study
most *Fusobacterium* sp.	Fus664	FITC	CTT GTA GTT CCG CYT ACC TC	52	No	40	46	[37]
*F. naviforme*, *F. nucleatum* subsp. *fusiforme*	Fnav1254	FITC	CTT CAC AGC TTT GCG ACT C	58	No	30	46	This study
*F. nucleatum*, *F. periodonticum*	Fnuc133c	FITC	GTT GTC CCT ANC TGT GAG GC	46	No	30	46	[37]
subgroup of the *Spirochaetaceae*	SPIRO1400	FITC	CTC GGA TGG TGT GAC GGG CG	60	No	ND	50	[38]
*T. medium* and *T. denticola*	TrepG1	Cy3	GAT TCC ACC CCT ACA CTT	58	No	20–50	46	This study
*T. denticola*	Td469	FITC	CAT GAC TAC CGT CAT CAA AGA AGC	56	No	ND	50	[39]
*A. actinomycetemcomitans*	Aa829	FITC	GGG CTA AAC CCC AAT CCC	53	No	ND	50	This study
*Campylobacter* sp.	Camp655	FITC	CAT CTG CCT CTC CCT YAC	57	No	30	46	[40]
*Cytophaga-Flavobacterium-Bacteroides cluster*	CFB935	Cy3	CCA CAT GTT CCT CCG CTT GT	54	No	>40	50	[38]
*Bacteroidaceae and Prevotellaceae*	BAC303	Cy3	CCA ATG TGG GGG ACC TT	49	No	0	50	[41]
*Prevotella* sp.	Prev394	FITC	GCA CGC TAC TTG GCT GG	56	No	25	46	[17]
*P. intermedia*	Pi425	FITC	CTT TAC TCC CCA ACA AAA GCA GTT TAC AA	57	No	20	46	[42]
*P. nigrescens*	Pnig657	Cy3	TCC GCC TGC GCT GCG TGT A	64	No	≤40	46	[43]
*T. forsythia*	Tfor582	Cy3	GCG GAC TTA ACA GCC CAC CT	64	No	40	46	[44]
*T. forsythia*	Tfor440	Cy3	CGT ATC TCA TTT TAT TCC CCT GTA	52	No	20	46	[42]
*T. forsythia*	Tfor127	Cy3	CTC TGT TGC GGG CAG GTT AC	65	No	40	46	[44]
*T. forsythia*	Tfor997	Cy3	TCA CTC TCC GTC GTC TAC	56	No	40	46	[44]
*P.gingivalis*	Pg477	FITC	CAA TAC TCG TAT CGC CCG TTA TTC	56	No	20	46	[42]
*P. endodontalis*	Pendo740	Cy3	CAG TGT CAG ACG GAG CCT	58	No	30–40	46	This study
*Capnocytophaga genus*	Capno371	FITC	TCA GTC TTC CGA CCA TTG	54	No	0	50	[45]

New probes developed in the present study have been designed with the ARB software package [46] and tested against a panel of reference strains for specificity (ACT218, Aa829, LAB759, Sel1469 and Fnav1254) or tested on subgingival plaque samples at increasing formamide concentrations to define assay conditions for maximum stringency and optimal specificity (TrepG1, Pendo740 and SynA1409). Probe LAB759 showed cross-reactivity with *Eikenella corrodens* and not identified cocci. In the present study, only rods gave a positive signal with probe LAB759.
